# Oculomotor Nerve Palsy as a Manifestation of Immune Thrombocytopenic Purpura: A Case Report

**DOI:** 10.7759/cureus.29723

**Published:** 2022-09-29

**Authors:** Andrew Manfra, Kyaw M Tun, Mark J Chang, Sandhya Wahi-Gururaj

**Affiliations:** 1 Internal Medicine, Kirk Kerkorian School of Medicine at the University of Nevada, Las Vegas, Las Vegas, USA

**Keywords:** microhemorrhage, thrombocytopenia, immune-hematology, oculomotor nerve (cn iii) palsy, itp

## Abstract

Immune thrombocytopenic purpura (ITP) is caused by alterations in the immune system resulting in platelet destruction. It often manifests clinically with bleeding or on routine lab work revealing thrombocytopenia in asymptomatic individuals. Neurologic manifestations of this condition are sparsely documented in the literature. Among the symptoms reported were case reports of ischemic strokes, transient ischemic attacks, mononeuropathy multiplex, and polyneuropathy as neurological complications from immune thrombocytopenic purpura. Isolated cranial nerve palsies are uncommon. The following case describes a patient with immune thrombocytopenic purpura who presented with an isolated cranial nerve III palsy. A 55-year-old presented with pain in the right eye that was found in a downward and lateral gaze paralysis. There was no evidence of central or peripheral neurovascular etiology on imaging. However, workup revealed isolated thrombocytopenia with platelets <2000/ml^3^. Other possible etiologies, such as human immunodeficiency virus (HIV) and infectious etiologies, were evaluated and excluded. Thrombotic thrombocytopenia purpura was excluded with the results from ADAMTS13 (a disintegrin and metalloproteinase with a thrombospondin type 1 motif, member 13*) *activity. The patient was appropriately transfused with platelets and was treated with methylprednisolone, which improved his platelets. At the time of discharge, the patient continued to have cranial nerve III palsy and was referred to follow up with hematology on an outpatient basis. In prior case reports where ITP presented as neurological deficits, there was evidence of intraneural microhemorrhage. Our case is unique in that the primary neurologic presentation without central nervous system pathology eventually led to the diagnosis of ITP. The symptoms were attributed to microhemorrhages that were not detected in imaging studies. Further studies are warranted to explore any correlation or causative association between ITP and neurological symptoms. This case report highlights the need to consider uncommon but possible manifestations of conditions that may initially appear seemingly irrelevant to the patient’s chief complaint.

## Introduction

Immune thrombocytopenic purpura (ITP) is a complex phenomenon defined as an isolated thrombocytopenia without anemia or leukopenia and without another apparent cause of thrombocytopenia. While there are multiple mechanisms associated with the pathophysiology of ITP, the thrombocytopenia observed in primary ITP is generally considered to be related to alterations in the immune system. Some suggested mechanisms involve somatic mutations in GII/IIIb platelet surface glycoproteins causing platelet antibodies, leading to increased platelet destruction through phagocytosis, complement activation, lysis, and decreased platelet production [1-4]. ITP is typically treated with glucocorticoids or intravenous immune globulin [1]. Patients have varying responses to treatments, highlighting the complex pathophysiology of ITP with regard to loss of immune tolerance toward platelet antigens and impaired thrombopoiesis. Clinical manifestations of ITP often present with malfunction of primary hemostasis leading to bleeding; however, neurological manifestations in ITP without clinically significant bleeding are rare. Immune-mediated neuropathy and intraneural hemorrhage have rarely been reported [5-12]. The following case describes a patient with ITP who presents with an isolated cranial nerve III palsy. 

## Case presentation

**Figure 1:**
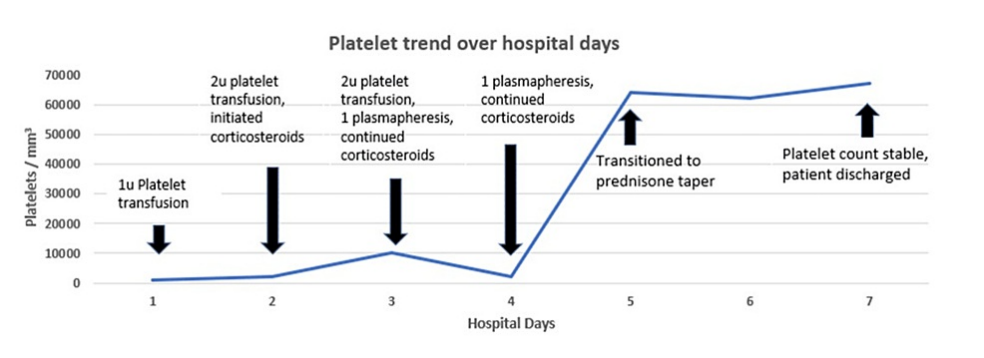
The graph displays the response to treatment course. Platelet transfusions, corticosteroids, and ultimately plasmapheresis were administered while awaiting confirmatory laboratory testing.

A 55-year-old man with a remote history of thrombocytopenia and essential hypertension was referred to the emergency department for severe thrombocytopenia and isolated right-eye cranial nerve III palsy. The patient’s eye symptoms developed three weeks prior and began with a non-radiating constant drilling pain behind the right eye. The next day his eye was paralyzed in a downward and lateral gaze. His vision was intact, and his left eye was unaffected. Upon further questioning, he reported a history of thrombocytopenia discovered during routine labs 10 years prior, for which he did not receive further evaluation or continued monitoring of the platelet level. Furthermore, the patient noted recurrent episodes of easy bruising and epistaxis lasting two to three days for many years. During this admission, physical examination was significant for a right eye abducted and deviated downwards, consistent with an isolated cranial nerve III palsy. Laboratory data was noteworthy for isolate severe thrombocytopenia with platelets <2000/ml3. The remainder of his evaluation was normal, including the white blood cell count, hemoglobin, complete metabolic profile, glycosylated hemoglobin, lipid panel, and thyroid-stimulating hormone. Due to the presence of his neurologic findings, the predominant differential diagnosis on admission included ITP versus thrombotic thrombocytopenic purpura (TTP). Peripheral blood smear demonstrated marked thrombocytopenia, with an absence of circulating immature blood cells, platelet clumping, and schistocytes. Other possible etiologies of thrombocytopenia were investigated and excluded, including human immunodeficiency virus, hepatic diseases, infection, and iatrogenic causes such as pharmacotherapy. ITP consequently remained as the diagnosis of exclusion. Nonetheless, the patient’s platelet count, hemolysis variable, absence of active cancer, absence of stem-cell or a solid-organ transplant, mean corpuscular volume, international normalized ratio, creatinine (PLASMIC) score was six based on platelet count of less than 2000 platelets/ml3, absence of hemolysis based on normal haptoglobin and bilirubin, absence of active cancer, absence of stem-cell or a solid-organ transplant, mean corpuscular volume of 84.3 femtoliters, international normalized ratio of 1.01, and creatinine 0.75 mg/dl. Given the high risk of TTP based on the PLASMIC score, the patient received two sessions of plasmapheresis for possible TTP. Concurrently, he was also treated for ITP with glucocorticoids and was transfused with five units of platelets for thrombocytopenia while awaiting results of ADMATS13 (a disintegrin and metalloproteinase with a thrombospondin type 1 motif, member 13) activity. Disseminated intravascular coagulation (DIC) and TTP were ultimately ruled out with a normal coagulation profile, fibrinogen of 228mg/dL, and ADAMTS13 activity >91%, respectively. There was no evidence of central neurological pathology on magnetic resonance neuroimaging and vascular imaging. He then received three days of 1 mg/kg methylprednisolone twice daily with improvement in his platelet count. At the time of discharge, the patient continued to have stable cranial nerve III palsy. After the platelet count returned to an acceptable range at 62000/ml3, the patient was discharged with a one-month prednisone taper and outpatient referral to hematology. The different forms of treatment provided over the course of hospitalization are summarized in Figure 1.

## Discussion

ITP often presents clinically with bleeding or on routine lab work revealing thrombocytopenia, such as in this case. Prior case reports of neurologic manifestations in ITP have described ischemic strokes, transient ischemic attacks, mononeuropathy multiplex, and polyneuropathy [6-14]. There have been case reports of patients with ITP and neurological deficits found to have intraneural microhemorrhage on autopsy [5,10]. One case reported a patient with a third cranial nerve palsy and ITP, noted to have autopsy findings of intraneural microhemorrhage in the cavernous portion of the third cranial nerve [5]. Another patient with ITP presented with right median and peroneal nerve distribution weakness, with an autopsy revealing intraneural hemorrhages at multiple sites in the right median nerve without extra-neural hemorrhage [10]. Based on these case reports and after excluding other possible etiologies based on our workup, we postulated that the patient’s isolated cranial nerve III palsy was best explained by intraneural microhemorrhage not seen on imaging. We present this case because the patient presented with primary neurologic findings of an isolated cranial nerve III palsy without central nervous system pathology leading to the discovery of his severe thrombocytopenia and ultimate diagnosis of ITP.

## Conclusions

This case report highlights the need to consider uncommon but possible manifestations of conditions that may initially appear seemingly irrelevant to the patient’s chief complaint. Further observational studies to investigate an association between severe thrombocytopenia in ITP and neurological symptoms would be prudent in the future. 
